# Sex and Race Differences in Obesity-Related Genetic Susceptibility and Risk of Cardiometabolic Disease in Older US Adults

**DOI:** 10.1001/jamanetworkopen.2023.47171

**Published:** 2023-12-08

**Authors:** Hairui Yu, Nicole Armstrong, Greg Pavela, Kathryn Kaiser

**Affiliations:** 1 Department of Health Behavior, School of Public Health, University of Alabama at Birmingham; 2 Department of Family and Community Medicine, School of Medicine, University of Alabama at Birmingham; 3 Department of Epidemiology, School of Public Health, University of Alabama at Birmingham

## Abstract

**Importance:**

The fat mass and obesity–associated gene (*FTO*) is associated with obesity phenotypes, but the association is inconsistent across populations. Within-population differences may explain some of the variability observed.

**Objective:**

To investigate sex differences in the association between *FTO* single-nucleotide variants (SNVs) and obesity traits among self-identified non-Hispanic Black and non-Hispanic White US adults, to examine whether the SNVs were associated with cardiometabolic diseases, and to evaluate whether obesity mediated the association between *FTO* SNVs and cardiometabolic diseases.

**Design, Setting, and Participants:**

This cross-sectional study used data from the Reasons for Geographic and Racial Differences in Stroke (REGARDS) study, a US population-based cohort study with available genetic data (assayed in 2018) and phenotypic data at baseline (enrolled 2003-2007). Participants were aged 45 to 98 years at baseline. Data were analyzed from October 2021 to October 2022.

**Exposures:**

Eleven SNVs in the *FTO* gene present among both Black and White participants.

**Main Outcomes and Measures:**

Objectively measured obesity indicators (body mass index and waist-to-height ratio), objectively measured and/or self-reported cardiometabolic diseases (hypertension, stroke history, heart disease, and diabetes), and self-reported social-economic and psychosocial status.

**Results:**

A total of 10 447 participants (mean [SD] age, 64.4 [9.7] years; 5276 [55.8%] women; 8743 [83.7%] Black and 1704 [16.3%] White) were included. In the White group, 11 *FTO* SNVs were significantly associated with obesity, hypertension, and diabetes using linear models (eg, body mass index: β = 0.536; 95% CI, 0.197-0.875), but none of the *FTO* SNVs were associated with obesity traits in the Black group. White males had a higher risk of obesity while White females had a higher risk of hypertension and diabetes. However, 1 *FTO* SNV (rs1121980) was associated with a direct increase in the risk of heart disease in Black participants not mediated by obesity (c′ = 0.145 [SE, 0.0517]; *P* = .01).

**Conclusions and Relevance:**

In this cross-sectional study of obesity phenotypes and their association with cardiometabolic diseases, the tested *FTO* SNVs reflected sex differences in White participants. Different patterns of associations were observed among self-identified Black participants. Therefore, these results could inform future work discovering risk alleles or risk scores unique to Black individuals or further investigating genetic risk in all US residents.

## Key Points

**Question:**

Are there race and sex differences in the association of *FTO* single-nucleotide variants (SNVs) with obesity phenotypes and cardiometabolic disease among self-identified Black and White US residents?

**Findings:**

In this cross-sectional study of 10 447 participants, none of the tested *FTO* SNVs were significantly associated with the risk of obesity in Black participants, while 11 *FTO *SNVs were associated with the risk of obesity in White participants. White males had a higher risk of obesity than White females. The SNV rs1121980 was associated with increased risk of heart disease in Black participants, independent of obesity.

**Meaning:**

The findings of this study suggest that sex-specific mechanisms for obesity risk and cardiometabolic diseases may require further investigation to inform genetic risk, and these may differ by race.

## Teaser

This cross-sectional study investigates sex differences in the association between *FTO* single-nucleotide variants and obesity traits among self-identified non-Hispanic Black and non-Hispanic White US adults.

## Introduction

The fat mass and obesity–associated (*FTO *[OMIM 610966]) gene is a well-established obesity-associated gene identified in multiple genomewide association studies (GWAS) since 2007.^1^ Its strong associations with body mass index, body composition, waist circumference, and metabolism have drawn wide attention from researchers.^2,3^ *FTO* is extensively expressed in human fat tissues and skeletal muscles as well as the arcuate nucleus of the hypothalamus that controls energy balance and energy metabolism.^1,4^ The critical role of the *FTO* variants in energy intake, energy consumption, and obesity has been demonstrated in previous studies.^5,6^

The mechanistic pathways relative to the specific risk leading to obesity remain poorly understood.^7^ Many candidate genes are associated with obesity-related phenotypes, and sexual dimorphism has been demonstrated in human and animal models, not simply due to the presence of gonadal hormones but also to the sex chromosome complement.^8,9,10^ Further complicating potential risk factors for obesity beyond obesity genes are the inconsistently observed ranks of prevalence between men and women within the same racial category in the United States as seen over time in the National Health and Nutrition Examination Survey cohorts.^11,12^ Since the intervals when race data were reported separately, there are more than 10–percentage point gaps between men and women for obesity rates, but with Black women being higher than their male counterparts and White women being 10 or more percentage points lower than their male counterparts.^12^ Besides obesity phenotypes, other sex differences in risk factors for cardiovascular disease, such as blood lipid levels and glucose regulation, have been reported.^13,14^

While the number of possibly influential genes has continued to increase in the literature, FTO continues to be observed with some inconsistencies.^15,16^ For example, research on the association between a genetic risk score and BMI across 5 birth cohorts using the Health and Retirement Study revealed that an additional copy of the A allele or the *FTO* allele (rs1558902: A) was significantly associated with a 0.37 (95% CI, 0.21-0.54)–unit higher BMI among 7482 people with primarily European identity and a 1.03 (95% CI, 0.27-1.79)–unit higher BMI among 1306 people with primarily African identity.^17^ Another meta-analysis of GWASs among 20 cohorts of individuals with European identity reported that *FTO* (rs9936385) was significantly associated with BMI and whole-body lean mass.^18^

Socioeconomic, racial, and sex differences have been reported in gene-environment interactions with obesity, along with an increased risk of cardiometabolic diseases.^13,19^ Prior research concluded that socioeconomic status (income and/or education) was negatively associated with the obesity risk of adult women in developed countries.^19,20,21^ Stronger obesity-related genetic effects have been associated with lower socioeconomic groups, such as those with less income or educational attainment.^22^ Previous analysis from the Reasons for Geographic and Racial Differences in Stroke (REGARDS) study shows that excess biological and social risk factors associated with coronary heart disease events in Black individuals remain a public health problem, along with a higher risk for premature death.^23,24,25,26^ The psychological factors, such as depressive symptoms, also account for increased mortality across racial and income groups.^27,28^

*FTO* is one of the most commonly identified obesity-related genes (eTable 1 in Supplement 1), but prior studies have not included a wide range of racial groups or social backgrounds across the lifespan. We aimed to extend this literature using *FTO* single-nucleotide variants (SNVs) to evaluate sex differences in obesity phenotypes in participants who self-identify as non-Hispanic Black and non-Hispanic White individuals to bridge the research gap.

To extend these previous studies reporting racial disparities in the United States, 4 associations were examined. First, the association between *FTO* SNVs (11 effect alleles: rs1558902: A; rs1121980: A; rs17817449: G; rs8050136: A; rs9935401: A; rs3751812: T; rs9936385: C; rs9939609: A; rs9941349: T; rs9930506: G; and rs9922708: T) and obesity indicators (body mass index [BMI, calculated as weight in kilograms divided by height in meters squared] and waist-to-height ratio [WHtR]) was investigated, controlling for sex and race. Second, we examined whether the association between *FTO* SNVs and obesity indicators varies by sex, controlling for age, socioeconomic status (education level, income level), and psychosocial status (perceived stress, depressive symptoms) among Black and White individuals separately. Third, based on any observed significant associations in the previous examination, we tested whether there is an association between *FTO* SNVs and cardiometabolic diseases (hypertension, history of stroke, heart disease, and diabetes) and whether this association varies by sex. Finally, we examined whether the association between *FTO* SNVs and cardiometabolic disease is mediated by obesity indicators and whether the mediating effect varies by sex.

## Methods

### Participants

The Reasons for Geographic and Racial Differences in Stroke (REGARDS) study is a large, longitudinal, population-based cohort focusing on risk for stroke in older men and women, heavily sampling from the southeastern United States. A detailed description of the study and its measurements has been published.^29,30^ The study protocol for this research was approved by the institutional review board at University of Alabama at Birmingham, and all participants provided written informed consent. This report follows the Strengthening the Reporting of Genetic Association Studies (STREGA) reporting guidelines for cross-sectional studies of genetic association studies.^31^ REGARDS staff enrolled 30 239 unrelated non-Hispanic Black and non-Hispanic White participants, aged 45 years and older at baseline, with oversampling of Black individuals and residents of the southeastern US. Sociodemographic, psychological, and medical history data were collected using a computer-assisted telephone interview at baseline. During an in-home examination, researchers measured anthropometrics, conducted a medication inventory, and collected blood and urine specimens.

The subsample that made up the genotyped cohort (10 543 with complete baseline data) was reviewed for obesity indicators, ie, BMI and WHtR, for the present analysis. Participants with extreme values in obesity indicators (BMI ≤18.5 or ≥40 [10.29%]; waist circumference [WC] ≤60 or ≥180 cm [0.91%]) were excluded.^32^ The final analysis cohort was identified, with 9458 with BMI and 10 447 with WHtR. eFigure 1 in Supplement 1 illustrates the cohort selection process.

### Genetic Assessment

The genetic exposures were the 11 available *FTO* SNVs located in the *FTO* gene that met the 5% minor allele frequency (MAF) and imputation quality (0.3) threshold and were present in Black and White strata (rs1558902: A; rs1121980: A; rs17817449: G; rs8050136: A; rs9935401: A; rs3751812: T; rs9936385: C; rs9939609: A; rs9941349: T; rs9930506: G; and rs9922708: T) (eTable 1 in Supplement 1). All SNVs in the study population did not deviate from the Hardy-Weinberg equilibrium. LDmatrix was used to determine linkage disequilibrium (LD) among the 11 SNVs by self-identified race group.^33^ The eAppendix in Supplement 1 provides details of genotyping methods and quality control. LD blocks by race group were created based on an LD threshold of 0.8. For significant LD block proxy SNVs in the models for obesity traits, we used those for mediation analysis of cardiovascular disease status at baseline in an exploratory analysis, as defined later.

### Study Outcomes

The primary outcomes of this study were obesity indicators and cardiometabolic disease. The obesity indicators based on researcher measured height, weight, and waist circumference were BMI and WHtR.^34,35^ Aligned with REGARDS protocol, the variables of cardiometabolic diseases were defined as current hypertension (systolic blood pressure ≥130 mm Hg or diastolic blood pressure ≥80 mm Hg or self-reported use of medication to control blood pressure), stroke (baseline self-reported stroke history), heart disease (self-reported history of myocardial infarction, coronary artery bypass graft, angioplasty, or stenting or evidence of myocardial infarction via electrocardiogram), and type 2 diabetes defined as fasting glucose level of 126 mg/dL or greater; nonfasting glucose, 200 mg/dL or greater, or self-reported use of pills or insulin to manage blood glucose (to convert glucose to millimoles per liter, multiply by 0.0555).

### Covariates

Participants self-reported sex (female and male), race (self-identified Black or White), and age at baseline. Black and White were the only categories available to participants in the REGARDS study. Self-reported income (annual household categories: <$20 000, $21 000-$34,000, $35 000-$74,000, or ≥$75 000), education levels (highest grade or year of school completed: <high school, high school, some college, or ≥college), perceived stress (Perceived Stress Scale),^36^ and depression (Center for Epidemiologic Studies Depression Scale)^37^ were included as covariates in the tested models.

### Statistical Analysis

Descriptive statistics were analyzed for each race and sex group. All continuous variables were evaluated for normality of distribution and multicollinearity. Baseline obesity phenotypes, age, and psychosocial status variables were compared between race and sex groups using analysis of variance and χ^2^ tests. The significance level was assessed by using the Bonferroni correction (α = .05 / No. of independent tests).^38^ All analyses were conducted using SAS version 9.4 (SAS Institute) and SPSS version 28.0 (IBM Corp).

Multiple linear regression analysis was performed to examine the associations between the explanatory variables and the obesity indicators (BMI and WHtR) in each race group. For significant SNVs identified from the main models, an interaction term (the product of sex and number of copies of *FTO* SNVs) was calculated and tested separately in each race group with multiple linear regressions. Based on significant associations identified in regression models, per methods of Hayes,^39^ mediation and moderation analyses with bootstrapping (5000 bootstrap samples) were performed to test whether, for each race group, an obesity indicator mediated the association between *FTO* SNVs and cardiometabolic diseases, which was potentially moderated by sex. Any with significant associations were evaluated in mediation models to investigate whether obesity played a role in indirect effects between sex groups. To augment power to detect genuine associations,^40,41,42^ the top 10 genetic principal components (PC 1 to PC 10) were included in each model to account for genetic ancestry.^43,44^ Based on LD patterns of the 11 *FTO* SNVs in each race group, statistical significance assessment was adjusted using a Bonferroni correction for 6 LD blocks, while in the White stratum, all 11 FTO variants were strongly linked (*r*^2^ > 0.8). The α level for Black participants was calculated as .05 / 6 independent tests, or .00833, while the α for White participants was .05 to assess statistical significance.

## Results

At enrollment, the overall mean (SD) age was 64.4 (9.7) years, there were 5276 (55.8%) women, 8743 (83.7%) Black participants, and 1704 (16.3%) White participants. See Table 1 for the mean differences in obesity indicators between race and sex groups. eTable 2 in Supplement 1 shows the frequency of the 11 *FTO* SNVs between race groups. There was a significant difference (>20%) in SNV frequencies for rs1558902: A, rs3751812: T, rs9941349: G, rs9930506: G, and rs9922708: T between the Black and White groups. However, all were greater than 10% MAF, so these differences would not affect statistical power substantially. Black participants had significantly higher BMI (difference, 1.6 [95% CI, 1.4-1.8]; *P* < .001) and WHtR (difference, 0.72 [95% CI, 0.68-0.76]; *P* < .001) compared with White participants.

**Table 1.** Comparisons of Obesity Indicators Between Sex and Self-Identified Race Groups by Analysis of Variance, Excluding Cases Analysis by Analysis

Phenotype	Race		F	*P* value^a^	Sex		F	*P* value^a^
	Black	White			Female	Male		
**BMI**								
No.	7382	1626	NA	NA	5276	4182	NA	NA
Mean (SD) [range]	29.50 (4.86) [18.51-39.99]	27.90 (4.54) [18.52-39.87]	149.09	<.001	29.90 (5.02) [18.51-39.99]	28.40 (4.48) [18.52-39.99]	231.56	<.001
**WHtR**								
No.	8743	1704	NA	NA	6043	4404	NA	NA
Mean (SD) [range]	0.58 (0.09) [0.35-1.13]	0.57 (0.08) [0.36-0.97]	51.86	<.001	0.59 (0.96) [0.36-1.13]	0.56 (0.78) [0.35-0.97]	274.99	<.001

Abbreviations: BMI, body mass index (calculated as weight in kilograms divided by height in meters squared); NA, not applicable; WHtR, waist to height ratio.

^a^ Welch tests performed for groups with unequal variances based on results from Levene tests.

Females had significantly higher BMI (difference, 1.5 [95% CI, 1.3-1.7]; *P* < .001) and WHtR (difference, 0.028 [95% CI, 0.025-0.032]; *P* < .001) compared with males. Compared with White participants, Black participants were significantly younger (difference, 4.78 [95% CI, 4.25-5.30] years; *P* < .001), reported higher stress and depression scores, and had lower education and income levels (eTables 3 and 4 in Supplement 1). Between sex groups, women were younger (difference, 1.09 [95% CI, 0.72-1.46] years; *P* < .001); reported higher stress and depression scores; and had lower-income levels (eTables 3 and 4 in Supplement 1).

eTables 5 and 6 in Supplement 1 provide results for LD for White participants and Black participants, for LD values and LD blocks, respectively, formed from these associations. Due to significant LD values observed among the 11 SNVs, we selected a proxy SNV to represent others, where the LD values were greater than 0.80. Thus, 6 proxy SNVs from the LD blocks were used in the adjusted regression models for Black participants (rs1558902, rs1121980, rs17817449, rs8050136, rs9941349, and rs9930506) and 1 for White participants (rs1558902). All proxy SNVs were used for the mediation analysis for Black (6 SNVs) and White (1 SNV) participants.

### Results of Regression Models

For White participants, all 11 *FTO* SNVs were significantly and positively associated with BMI and WHtR (eTable 7 in Supplement 1). In contrast, results of the unadjusted models show that only 2 of 6 *FTO* LD block SNVs were significantly and positively associated with BMI in Black participants.

In adjusted models using age, education, income, perceived stress, and depressive symptoms as covariates (Table 2), none of the LD block SNVs were associated with an obesity indicator in Black participants. All 11 *FTO* SNVs remained associated with BMI in White participants (β = 0.536 [95% CI, 0.197-0.875]) and WHtR (β = 0.007 [95% CI, 0.001-0.013]). Table 3 presents the adjusted models summary. Older age was significantly associated with lower BMI but significantly associated with higher WHtR in each race group. Education levels were significantly negatively associated with BMI and WHtR in each race group. In Black participants, depression was significantly positively associated with WHtR. Only in White participants were significant interactions between *FTO* and sex as well as *FTO* and age identified for BMI and WHtR (eTables 8 and 9 in Supplement 1). For example, with rs1558902: A, the coefficient for BMI was higher for men (β = 0.781; SE, 0.192; *P* < .001).

**Table 2.** Associations of BMI and WHtR With *FTO* SNVs, Controlling for Sex and Psychosocial Status in Self-Identified Black Participants^a^

*FTO* SNV LD block proxy	*FTO*		Sex		Age		Education		Income		Stress		Depression	
	β (SE)	*P* value	β (SE)	*P* value	β (SE)	*P* value	β (SE)	*P* value	β (SE)	*P* value	β (SE)	*P* value	β (SE)	*P* value
BMI^b^														
rs1558902	0.272 (0.132)	.04	−1.807 (0.121)	<.001	−0.061 (0.007)	<.001	−0.287 (0.064)	<.001	0.199 (0.072)	.006	−0.028 (0.024)	.25	0.042 (0.03)	.16
rs1121980	0.093 (0.084)	.27	−1.805 (0.122)	<.001	−0.061 (0.007)	<.001	−0.287 (0.064)	<.001	0.199 (0.072)	.006	−0.028 (0.024)	.25	0.042 (0.03)	.16
rs17817449	0.041 (0.085)	.63	−1.807 (0.122)	<.001	−0.061 (0.007)	<.001	−0.287 (0.064)	<.001	0.199 (0.072)	.006	−0.028 (0.024)	.25	0.042 (0.03)	.17
rs8050136	0.054 (0.084)	.52	−1.807 (0.122)	<.001	−0.061 (0.007)	<.001	−0.287 (0.064)	<.001	0.199 (0.072)	.006	−0.028 (0.024)	.25	0.042 (0.03)	.16
rs9941349	0.222 (0.106)	.04	−1.806 (0.122)	<.001	−0.061 (0.007)	<.001	−0.287 (0.064)	<.001	0.199 (0.072)	.006	−0.028 (0.024)	.25	0.042 (0.03)	.16
rs9930506	0.184 (0.101)	.07	−1.807 (0.122)	<.001	−0.061 (0.007)	<.001	−0.287 (0.064)	<.001	0.199 (0.072)	.006	−0.028 (0.024)	.25	0.042 (0.03)	.16
WHtR^c^														
rs1558902	0.003 (0.002)	.21	−0.021 (0.002)	<.001	0.001 (<0.001)	<.001	−0.006 (0.001)	<.001	−0.002 (0.001)	.05	−0.001 (<0.001)	.23	0.002 (<0.001)	<.001
rs1121980	0.001 (0.002)	.38	−0.021 (0.002)	<.001	0.001 (<0.001)	<.001	−0.006 (0.001)	<.001	−0.002 (0.001)	.05	−0.001 (<0.001)	.23	0.002 (<0.001)	<.001
rs17817449	0.001 (0.002)	.94	−0.021 (0.002)	<.001	0.001 (<0.001)	<.001	−0.006 (0.001)	<.001	−0.002 (0.001)	.05	−0.001 (<0.001)	.23	0.002 (<0.001)	<.001
rs8050136	0.001 (0.002)	.60	−0.021 (0.002)	<.001	0.001 (<0.001)	<.001	−0.006 (0.001)	<.001	−0.002 (0.001)	.05	−0.001 (<0.001)	.23	0.002 (<0.001)	<.001
rs9941349	0.002 (0.002)	.19	−0.021 (0.002)	<.001	0.001 (<0.001)	<.001	−0.006 (0.001)	<.001	−0.002 (0.001)	.05	−0.001 (<0.001)	.24	0.002 (<0.001)	<.001
rs9930506	0.003 (0.002)	.06	−0.021 (0.002)	<.001	0.001 (<0.001)	<.001	−0.006 (0.001)	<.001	−0.002 (0.001)	.05	−0.001 (<0.001)	.23	0.002 (<0.001)	<.001

Abbreviation: BMI, body mass index; LD, linkage disequilibrium; SNV, single-nucleotide variant; WHtR, waist-to-height ratio.

^a^ Female was a reference group for sex, and lower education and income level was the reference group from psychosocial status. The significance level was assessed at .05.

^b^ General linear model: BMI = intercept + SNV (eg, LD Block 1: rs1558902: A) + sex + age + income + education + stress + depression + PC 1-10 + error.

^c^ General linear model: WHtR = intercept + SNV (eg, LD Block 1: rs1558902: A) + sex + age + income + education + stress + depression + PC 1-10 + error.

**Table 3.** Associations of BMI and WHtR With *FTO* SNV rs1558902, Controlling for Sex and Psychosocial Status in Self-Identified White Participants

*FTO* SNV LD block proxy	*FTO*		Sex		Age		Education		Income		Stress		Depression	
	β (SE)	*P* value	β (SE)	*P* value	β (SE)	*P* value	β (SE)	*P* value	β (SE)	*P* value	β (SE)	*P* value	β (SE)	*P* value
BMI^b^														
rs1558902	0.536 (0.173)	.002	0.525 (0.256)	.04	−0.082 (0.012)	<.001	−0.400 (0.136)	.003	−0.042 (0.149)	.78	0.007 (0.056)	.91	0.010 (0.067)	.88
WHtR^c^														
rs1558902	0.007 (0.003)	.02	0.013 (0.005)	.004	0.001 (<0.001)	.04	−0.009 (0.002)	<.001	−0.006 (0.003)	.03	0.001 (0.001)	.61	0.002 (0.001)	.07

Abbreviation: BMI, body mass index; LD, linkage disequilibrium; SNV, single-nucleotide variant; WHtR, waist-to-height ratio.

^a^ Female was a reference group for sex, and lower education and income level was the reference group from psychosocial status. The Bonferroni-correct significance level was .05.

^b^ General linear model: BMI = intercept + SNV (proxy LD block SNV: rs1558902) + sex + age + income + education + stress + depression + PC 1-10 + error.

^c^ General linear model: WHtR = intercept + SNV (proxy LD block SNV: rs1558902) + sex + age + income + education + stress + depression + PC 1-10 + error.

### Results of Mediation Analysis for Cardiovascular Disease Associations by Race and Sex

Given the initial model results and commonly observed associations of BMI and WHtR with cardiometabolic disease, we explored whether these associations might differ between race and sex groups. In the mediation and moderation analyses (Tables 4 and 5; eFigure 2 in Supplement 1), only *FTO* LD block 2 had significant direct effects on heart disease among Black participants (c′ = 0.145 [SE, 0.0517]; *P* = .01), not mediated through BMI or WHtR. The direct effect did not vary by sex (*P* = .76) (eTable 10 in Supplement 1). There were positive (but not significant) indirect effects of *FTO* through obesity indicators and direct effects of *FTO* on other cardiometabolic diseases in Black participants (Table 4). In White participants, *FTO* had significant indirect effects via obesity indicators on hypertension (Table 4; eFigure 3A in Supplement 1) (eg, via WHtR: a = 0.0071 [SE, 0.0028]; *P* = .01; b = 7.9672 [SE, 0.9951]; *P* < .001) and diabetes (eTable 11 and eFigure 3B in Supplement 1) (eg, via WHtR: a, 0.0062 [SE, 0.0028]; *P* = .03; b, 14.60 [SE, 3.79]; *P* < .001), which differed by sex (eg, via WHtR on hypertension, index = −0.026; via WHtR on diabetes, index = −0.016) (eTable 11 in Supplement 1).

**Table 4.** Results of Mediation and Moderation Analyses of BMI, WHtR, *FTO* (rs1121980), and Heart Disease in Self-Identified Black Participants and of BMI, WHtR, *FTO* (rs1558902), and Hypertension Among Self-Identified White Partcipants^a^

Model	Consequents			
	Path M (obesity indicator)		Path Y (outcome)	
	Coefficient (SE)	*P *value	Coefficient (SE)	*P *value
**Model BMI-heart disease among Black participants**				
Antecedent				
X, rs1121980	a, 0.0852 (0.0841)	.31	c′, 0.1450 (0.0517)	.01
M, BMI	NA	NA	b, 0.0212 (0.0076)	.01
Constant	i_m_, 38.1841 (0.5852)	<.001	i_y_, −4.7185 (0.4622)	<.001
*R* ^2^	0.0529^b^	<.001	0.0451	<.001
**Model WHtR-heart disease among Black participants**				
Antecedent				
X, rs1121980	a, 0.0008 (0.0015)	.59	c′, 0.1149 (0.0493)	.02
M, BMI	NA	NA	b, 1.2860 (0.3871)	.001
Constant	i_m_, 0.6638 (0.0103)	<.001	i_y_, −4.7370 (0.4335)	<.001
*R* ^2^	0.0485^c^	<.001	0.0451	<.001
**Model BMI-hypertension among White participants**				
Antecedent				
X, proxy LD block SNV rs1558902	a, 0.5407 (0.1734)	.002	c′, −0.0276 (0.0940)	.77
M, BMI	NA	NA	b, 0.1168 (0.0157)	<.001
Constant	i_m_, 32.8935 (1.5525)	<.001	i_y_, −3.4320 (1.2979	.01
*R* ^2^	0.0588^d^	<.001	0.1519	<.001
**Model WHtR-hypertension among White participants**				
Antecedent				
X, proxy LD block SNV rs1558902	a, 0.0071 (0.0028)	.01	c′, −0.0175 (0.0944)	.85
M, WHtR	NA	NA	b, 7.9672 (0.9951)	<.001
Constant	i_m_, 0.5585 (0.0250)	<.001	i_y_, −4.1055 (1.2936)	.002
*R* ^2^	0.0548^e^	<.001	0.1602	<.001

Abbreviations: a, effect coefficient between genotype and obesity indicator; b, effect coefficient between mediator and outcome; BMI, body mass index, c′, direct coefficient between genotype and outcome; i_m_, intercept of mediator equation; i_y_, intercept of direct outcome equation; M, mediator tested; NA, not applicable; SNV, single-nucleotide variant; WHtR, waist-to-height ratio; X, genotype adjusted for ancestry principal components, age and psychosocial variables; Y, outcome.

^a^ Indirect effect is calculated as a × b; direct effect, c′; total effect, a × b + c′.

^b^ F = 21.3216.

^c^ F = 21.5925.

^d^ F = 4.9571.

^e^ F = 4.5871.

**Table 5.** Results of Moderation Analyses of BMI, WHtR, Hypertension, and Sex in Self-identified White Participants

Subgroup	Consequent			
	M (obesity indicator)		Y (hypertension)	
	Coefficient (SE)	*P* value	Coefficient (SE) [95% CI]	Index of moderated mediation
**Model BMI-hypertension **				
Female	0.1440 (0.0227)	<.001	0.0779 (0.0291) [0.0282-0.1422]	−0.0289
Male	0.0906 (0.0215)	<.001	0.0490 (0.0215) [0.0151-0.0037]	
**Model WHtR-hypertension**				
Female	9.4080 (1.2549)	<.001	0.0719 (0.0719) [0.0126-0.1398]	−0.0255
Male	6.0693 (1.3042)	<.001	0.0464 (0.0464) [0.0081-0.1022]	

Abbreviations: BMI, body mass index; M, mediator; WHtR, waist-to-height ratio; Y, outcome.

## Discussion

These results show important differences in genotype-phenotype association by sex among White participants, which have different patterns for Black participants. The often-reported *FTO* obesity risk alleles did not show the same strong associations for the Black group, and associations between obesity and cardiometabolic disease were different by sex for White individuals.

Many of the essential socioeconomic variables are consistent with prior investigations, which concluded that socioeconomic status (education level) was negatively associated with obesity risk for adults in developed countries.^20,21^ Depression was positively associated with obesity risk in this sample of older US residents. Notably, there was significantly higher risk of abdominal obesity and elevated visceral fat for Black women. Through physiological mechanisms, the hypothalamic-pituitary-adrenal (HPA) axis, the regulation of the metabolic pathways, or inflammation, the negative psychosocial status may interrupt energy metabolism and increase risk of abdominal obesity, an immuno-metabolic trait.^45,46^ Because of an age-related increase in visceral fat rather than subcutaneous or total body fat and a decrease in skeletal muscle indicating osteosarcopenic obesity,^47,48^ negative psychosocial statuses combined with a considerable decline in body composition may exacerbate health risks in vulnerable populations.^49,50,51^

For risks of obesity and cardiometabolic diseases, this study found that the degree of variance using *FTO* SNVs was different between self-identified race groups. Prior studies showed that Black individuals disproportionately experience heart disease mortality.^23,24^ A recent study highlighted the importance of exploring the circulating metabolome as a potential intermediary explaining differences between Black and White individuals regarding the associations between circulating metabolites and incident coronary heart disease.^52^ Our study indicated that independent of obesity phenotypes, *FTO* rs1121980: A had a significant direct association with heart disease in Black participants. Similar to prior studies, the significant association between rs1121980 and obesity was identified in a small West African sample but not in the Black sample^53^ and suggested a stronger association with obesity in White participants.^54^ In a GWAS study (GOLDN study on 707 US adults, not race specific), rs1121980 appeared to have important pleiotropic effects, with a significant direct association with high-density lipoprotein levels and an indirect effect on triglyceride levels.^55^ During early adipocyte differentiation and through a tissue-autonomous manner, the *FTO* allele associated with obesity could promote a reduction in mitochondrial thermogenesis, excessive accumulation of triglycerides, and an increase in lipid storage.^56^ Our results may identify more complete gene ontology pathways to understand the genes and variants that balance blood triglycerides as an important cardiovascular disease risk factor^57,58^ but showing disparities between White and Black adults.^59,60^

In contrast to the finding that no *FTO* variants were associated with obesity risk among Black participants, the 11 *FTO* SNVs examined here were significantly associated with obesity phenotypes (higher in White males) and risks of hypertension and diabetes (higher in White females) in the White group. Similar to previous findings in Europeans (490 Finnish participants), the AA genotype of rs9939609 was associated with an adjusted 2-fold increased risk (hazard ratio, 2.09; 95% CI, 1.17-3.73) of cardiovascular disease (morbidity and mortality) among male participants,^61^ and a higher incidence of coronary heart disease and cardiovascular disease events or death was associated with rs9939609 (AA genotype, OR = 1.90; *P* = .002 and *P* = .004, respectively) among 1200 Finnish people at baseline.^62^ Further, a meta-analysis of 1497 Black and West African adults concluded that *FTO*, along with *IRS1*, *KLF14*, and *PPARG*, played active roles via insulin resistance in the development of type 2 diabetes and other metabolic disorders.^63^ However, previous genetic risk score modeling (including rs9939609 as 1 of 46 gene variants) of 990 Black and 1644 White individuals aged 44 to 69 years found that cumulative allele load was associated with the risk of type 2 diabetes in White individuals, but only marginally in Black individuals.^64^

### Limitations

This study has limitations. The present analysis shows substantial differences between sex and self-identified race groups regarding obesity and cardiometabolic disease risk in this large national sample of 8743 Black and 1704 White individuals. However, we used only SNVs from 1 obesity risk gene (*FTO*) rather than some of the proposed genetic risk scores. For obesity and associated cardiometabolic diseases, the *FTO* SNVs may explain more of the variance in White individuals, or there may be other yet-to-be-identified risk or protective factors that were not included here. Participants in the REGARDS study were only allowed to self-identify as Black or White. These self-identifications do not preclude admixture in an individual’s ancestry. Additionally, we did not control for any potential familial relationships between participants, but these were screened out on enrollment in REGARDS.

## Conclusions

In this cross-sectional study of a large sample of US adults of self-identified African and European race groups, we observed that the often-reported *FTO* obesity risk alleles were not associated with obesity for the Black group while controlling for race and psychosocial variables and that association between obesity and cardiometabolic disease varied by sex for White participants with rs1558902. More studies should focus on discovering risk alleles or risk scores unique to Black individuals and exploring the influence of genotypes, including sex chromosome complement, to inform mechanisms in the prevention and management of obesity and cardiometabolic diseases. These data support future work refining genetic risk scores and personalized medicine for all US residents.

## Notes

Supplement 1. **eTable 1.** Selected Obesity-Related Human Studies That Investigated 11 FTO SNVs for Further Analyses **eAppendix.** Genotyping and Quality Control Details for REGARDS Participants **eFigure 1.** Study Participant Selection **eTable 2.** Comparisons of Effect Allele Frequencies of FTO SNVs Between Self-Identified Race Groups **eTable 3.** Comparisons of Baseline Age, Income, Education Levels, Depression Score, and Stress Score Between Sex and Self-Identified Race Groups for BMI Analysis **eTable 4.** Comparisons of Baseline Age, Income, Education Levels, Depression Score, and Stress Score Between Sex and Self-identified Race Groups for Waist-to-Height Ratio Analysis **eTable 5.** Results of Linkage Disequilibrium Among the 11 FTO SNVs in Self-Identified White Participants **eTable 6.** Results of Linkage Disequilibrium Among the 11 FTO SNVs in Self-Identified Black Participants **eTable 7.** Associations Between BMI and WHtR by FTO Proxy SNVs, Controlling for Sex (Females as the Reference Group) in Self-Identified Black Participants or White Participants **eTable 8.** Interaction Terms Between FTO SNVs, Sex, and Age in Black Participants, Controlling for Psychosocial Status (Lower Education Levels and Lower Income Levels as the Reference Group) **eTable 9.** Significant Interaction Terms Between FTO SNV rs1558902:A, Sex, and Age in White Participants, Controlling for Psychosocial Status (Lower Education Levels and Lower Income Levels as the Reference Group) **eFigure 2.** Mediation Model of Obesity Indicators (BMI) in Relation With 6 FTO LD Block Proxy SNVs and Heart Disease in Black Participants **eTable 10.** Interaction Terms Between Genetic Susceptibility by FTO SNV rs1121980: A and Sex in Black Participants **eFigure 3.** Mediation Model of Obesity Indicators (BMI and WHtR) in Relation With FTO SNVs, and Hypertension/Diabetes in White Participants **eTable 11.** Results of Mediation and Moderation analyses of BMI/WHtR, LD Block 1 (rs1558902:A), and Diabetes (No Diabetes as the Reference Group) in White Participants

Supplement 2.
Data Sharing Statement
