# Supplementary material for: Sex and Race Differences in Obesity-Related Genetic Susceptibility and Risk of Cardiometabolic Disease in Older US Adults
Source: JAMA Netw Open. 2023 Dec 8;6(12):e2347171. doi: 10.1001/jamanetworkopen.2023.47171 (PMC10709778; doi:10.1001/jamanetworkopen.2023.47171)
Supplement: Supplement 1. — eTable 1. Selected Obesity-Related Human Studies That Investigated 11 FTO SNVs for Further Analyses eAppendix. Genotyping and Quality Control Details for REGARDS Participants eFigure 1. Study Participant Selection eTable 2. Comparisons of Effect Allele Frequencies of FTO SNVs Between Self-Identified Race Groups eTable 3. Comparisons of Baseline Age, Income, Education Levels, Depression Score, and Stress Score Between Sex and Self-Identified Race Groups for BMI Analysis eTable 4. Comparisons of Baseline Age, Income, Education Levels, Depression Score, and Stress Score Between Sex and Self-identified Race Groups for Waist-to-Height Ratio Analysis eTable 5. Results of Linkage Disequilibrium Among the 11 FTO SNVs in Self-Identified White Participants eTable 6. Results of Linkage Disequilibrium Among the 11 FTO SNVs in Self-Identified Black Participants eTable 7. Associations Between BMI and WHtR by FTO Proxy SNVs, Controlling for Sex (Females as the Reference Group) in Self-Identified Black Participants or White Participants eTable 8. Interaction Terms Between FTO SNVs, Sex, and Age in Black Participants, Controlling for Psychosocial Status (Lower Education Levels and Lower Income Levels as the Reference Group) eTable 9. Significant Interaction Terms Between FTO SNV rs1558902:A, Sex, and Age in White Participants, Controlling for Psychosocial Status (Lower Education Levels and Lower Income Levels as the Reference Group) eFigure 2. Mediation Model of Obesity Indicators (BMI) in Relation With 6 FTO LD Block Proxy SNVs and Heart Disease in Black Participants eTable 10. Interaction Terms Between Genetic Susceptibility by FTO SNV rs1121980: A and Sex in Black Participants eFigure 3. Mediation Model of Obesity Indicators (BMI and WHtR) in Relation With FTO SNVs, and Hypertension/Diabetes in White Participants eTable 11. Results of Mediation and Moderation analyses of BMI/WHtR, LD Block 1 (rs1558902:A), and Diabetes (No Diabetes as the Reference Group) in White Participants [file jamanetwopen-e2347171-s001.pdf]

Supplemental Online Content

Yu H, Armstrong N, Pavela G, Kaiser K. Race and sex differences in obesity-related genetic susceptibility and risk of cardiometabolic disease in older adults. *JAMA Netw Open.* 2023;6(12):e2347171. doi:10.1001/jamanetworkopen.2023.47171

eTable 1. Selected Obesity-Related Human Studies That Investigated 11 FTO SNVs for Further Analyses

eAppendix. Genotyping and Quality Control Details for REGARDS Participants

eFigure 1. Study Participant Selection

eTable 2. Comparisons of Effect Allele Frequencies of FTO SNVs Between Self-Identified Race Groups

eTable 3. Comparisons of Baseline Age, Income, Education Levels, Depression Score, and Stress Score Between Sex and Race Groups for BMI Analysis

eTable 4. Comparisons of Baseline Age, Income, Education Levels, Depression Score, and Stress Score Between Sex and Self-Identified Race Groups for Waist-to-Height Ratio Analysis

eTable 5. Results of Linkage Disequilibrium Among the 11 FTO SNVs in Self-Identified White Participants

eTable 6. Results of Linkage Disequilibrium Among the 11 FTO SNVs in Self-Identified Black Participants

eTable 7. Associations Between BMI and WHtR by FTO Proxy SNVs, Controlling for Sex (Females as the Reference Group) in Self-Identified Black Participants or White Participants

eTable 8. Interaction Terms Between FTO SNVs, Sex, and Age in Black Participants, Controlling for Psychosocial Status (Lower Education Levels and Lower Income Levels as the Reference Group)

eTable 9. Significant Interaction Terms Between FTO SNV rs1558902:A, Sex, and Age in White Participants, Controlling for Psychosocial Status (Lower Education Levels and Lower Income Levels as the Reference Group)

eFigure 2. Mediation Model of Obesity Indicators (BMI) in Relation With 6 FTO LD Block Proxy SNVs and Heart Disease in Black Participants

eTable 10. Interaction Terms Between Genetic Susceptibility by FTO SNV rs1121980: A and Sex in Black Participants

eFigure 3. Mediation Model of Obesity Indicators (BMI and WHtR) in Relation With FTO SNVs, and Hypertension/Diabetes in White Participants

© 2023 Yu H et al. *JAMA Network Open.*

eTable 11. Results of Mediation and Moderation analyses of BMI/WHtR, LD Block 1 (rs1558902:A), and Diabetes (No Diabetes as the Reference Group) in White Participants

This supplemental material has been provided by the authors to give readers additional information about their work.

© 2023 Yu H et al. *JAMA Network Open.*

eTable 1. Selected Obesity-Related Human Studies That Investigated 11 *FTO* SNVs for Further Analyses

#	FTO SNVs	Studies	Study Participants	Study Designs	Relevant Outcomes
1	rs1558902	(Wang, 2018) [1]	8828 European American women and 5218 European American men	Prospective cohort	BMI
		(Bray, 2019) [2]	811 women and men, 80% with European identity	Randomized trail	Fat mass
2	rs1121980	(Wing, 2011) [3]	458 European Americans, 373 Latino Americans, and 288 African Americans	Cross-sectional	BMI and acute insulin response
		(Shimaoka, 2010) [4]	1514 Japanese adults	Cross-sectional	Plasma glucose and BMI
3	rs17817449	(Nock, 2011) [5]	759 European Americans and 469 African Americans	Cross-sectional	BMI
		(Wang, 2011) [6]	1060 European Americans	Genome-wide association	BMI
4	rs8050136	(Nock, 2011) [5]	759 European Americans and 469 African Americans	Cross-sectional	BMI
		(Wing, 2011) [3]	458 European Americans, 373 Latino Americans, and 288 African Americans	Cross-sectional	BMI and acute insulin response
5	rs9935401	(Holzapfel, 2011) [7]	6251 men and 6174 women in German	Cross-sectional	BMI
		(Olza, 2013) [8]	534 children in Spain	Case-control	BMI and CVD biomarkers
6	rs3751812	(Liaw, 2019) [9]	10,832 adults in Taiwan	Cross-sectional	BMI
		(Hassanein, 2010) [10]	10819 African Americans	Cross-sectional	BMI
7	rs9936385	(Zillikens, 2017) [11]	38,292 adults with European identity	Meta-analysis	Lean body mass
		(Minohara, 2021) [12]	176 Japanese adults	Cross-sectional	Lean body mass
8	rs9939609	(Kilpeläinen, 2011) [13]	218,166 adults and 19,268 children, 95% with European identity	Meta-analysis	BMI, WC, and fat percent
		(Naja, 2021) [14]	194 Emirati adults	Cross-sectional	BMI
9	rs9941349	(Hassanein, 2010) [10]	10819 African Americans	Cross-sectional	BMI
		(Yako, 2015) [15]	560 adults in South Africa with mixed identity	Cross-sectional	BMI and type 2 diabetes
10	rs9930506	(Goutzelas, 2017) [16]	203 Greek adults	Cross-sectional	BMI
		(Naja, 2021) [14]	194 Emiratis	Cross-sectional	BMI
11	rs9922708	(McCaffery, 2013) [17]	1595 European Americans and 324 African Americans	Randomized trail	Weight
		(Helgeland, 2019) [18]	9286 Norwegian children	Genome-wide association	BMI

© 2023 Yu H et al. *JAMA Network Open.*

REFERENCES for eTable 1

1. Wang T, Heianza Y, Sun D, et al. Improving adherence to healthy dietary patterns, genetic risk, and long term weight gain: gene-diet interaction analysis in two prospective cohort studies. *BMJ*. Jan 10 2018;360:j5644. doi:10.1136/bmj.j5644

2. Bray GA, Krauss RM, Sacks FM, Qi L. Lessons Learned from the POUNDS Lost Study: Genetic, Metabolic, and Behavioral Factors Affecting Changes in Body Weight, Body Composition, and Cardiometabolic Risk. *Curr Obes Rep*. Sep 2019;8(3):262-283. doi:10.1007/s13679-019-00353-1

3. Wing MR, Ziegler JM, Langefeld CD, et al. Analysis of FTO gene variants with obesity and glucose homeostasis measures in the multiethnic Insulin Resistance Atherosclerosis Study cohort. *Int J Obes (Lond)*. Sep 2011;35(9):1173-82. doi:10.1038/ijo.2010.244

4. Shimaoka I, Kamide K, Ohishi M, et al. Association of gene polymorphism of the fat-mass and obesity-associated gene with insulin resistance in Japanese. *Hypertens Res*. Mar 2010;33(3):214-8. doi:10.1038/hr.2009.215

5. Nock NL, Plummer SJ, Thompson CL, Casey G, Li L. FTO polymorphisms are associated with adult body mass index (BMI) and colorectal adenomas in African-Americans. *Carcinogenesis*. May 2011;32(5):748-56. doi:10.1093/carcin/bgr026

6. Wang K, Li WD, Zhang CK, et al. A genome-wide association study on obesity and obesity-related traits. *PLoS One*. Apr 28 2011;6(4):e18939. doi:10.1371/journal.pone.0018939

7. Holzapfel C, Grallert H, Baumert J, et al. First investigation of two obesity-related loci (TMEM18, FTO) concerning their association with educational level as well as income: the MONICA/KORA study. *Journal of Epidemiology and Community Health (1979-)*. 2011;65(2):174-176.

8. Olza J, Ruperez AI, Gil-Campos M, et al. Influence of FTO variants on obesity, inflammation and cardiovascular disease risk biomarkers in Spanish children: a case-control multicentre study. *BMC Med Genet*. Dec 1 2013;14:123. doi:10.1186/1471-2350-14-123

9. Liaw YC, Liaw YP, Lan TH. Physical Activity Might Reduce the Adverse Impacts of the FTO Gene Variant rs3751812 on the Body Mass Index of Adults in Taiwan. *Genes (Basel)*. May 9 2019;10(5):354. doi:10.3390/genes10050354

10. Hassanein MT, Lyon HN, Nguyen TT, et al. Fine mapping of the association with obesity at the FTO locus in African-derived populations. *Hum Mol Genet*. Jul 15 2010;19(14):2907-16. doi:10.1093/hmg/ddq178

11. Zillikens MC, Demissie S, Hsu YH, et al. Large meta-analysis of genome-wide association studies identifies five loci for lean body mass. *Nat Commun*. Jul 19 2017;8(1):80. doi:10.1038/s41467-017-00031-7

12. Minohara T, Noso S, Babaya N, et al. Associations between genetic loci related to lean mass and body composition in type 2 diabetes patients. *Geriatrics & Gerontology International*. 08/01 2021;21doi:10.1111/ggi.14259

13. Kilpeläinen TO, Qi L, Brage S, et al. Physical activity attenuates the influence of FTO variants on obesity risk: a meta-analysis of 218,166 adults and 19,268 children. *PLoS medicine*. 2011;8(11):e1001116-e1001116. doi:10.1371/journal.pmed.1001116

14. Naja F, Itani L, Hammoudeh S, et al. Dietary Patterns and Their Associations With the FTO and FGF21 Gene Variants Among Emirati Adults. *Front Nutr*. 2021;8:668901. doi:10.3389/fnut.2021.668901

© 2023 Yu H et al. *JAMA Network Open.*

15. Yako YY, Madubedube JH, Kengne AP, Erasmus RT, Pillay TS, Matsha TE. Contribution of ENPP1, TCF7L2, and FTO polymorphisms to type 2 diabetes in mixed race ethnic population of South Africa. *Afr Health Sci*. Dec 2015;15(4):1149-60. doi:10.4314/ahs.v15i4.14

16. Goutzelas Y, Kotsa K, Vasilopoulos Y, et al. Association analysis of FTO gene polymorphisms with obesity in Greek adults. *Gene*. May 20 2017;613:10-13. doi:10.1016/j.gene.2017.02.033

17. McCaffery JM, Papandonatos GD, Huggins GS, et al. FTO predicts weight regain in the Look AHEAD clinical trial. *Int J Obes (Lond)*. Dec 2013;37(12):1545-52. doi:10.1038/ijo.2013.54

18. Helgeland O, Vaudel M, Juliusson PB, et al. Genome-wide association study reveals dynamic role of genetic variation in infant and early childhood growth. *Nat Commun*. Oct 1 2019;10(1):4448. doi:10.1038/s41467-019-12308-0

© 2023 Yu H et al. *JAMA Network Open.*

eAppendix. Genotyping and Quality Control Details for REGARDS Participants

An initial subset of 10,656 REGARDS participants (8,916 AA and 1,740 EA) underwent genotyping using Illumina Infinium Multi-Ethnic AMR/AFR BeadChip arrays (Illumina, Inc, San Diego, CA) as previously described [1,2]. Quality control at both the sample and variant levels was performed. Samples were removed if they were internal duplicates, HapMap controls, sex mismatches, or exhibited a high proportion of missing variants [1,2]. Genotype imputation was performed using the BioData Catalyst imputation server and the Trans-omics for Precision Medicine (TOPMed) release 2 (Freeze 8) reference panel [3]. Principal component analysis was performed using EIGENSTRAT SmartPCA software, and individuals were considered outliers and removed from subsequent analysis if they were outside of six standard deviations [4]. PLINK version 1.9 was used to code the genotypes as additive allele dosages for the *FTO* SNPs [5].

References

1. Armstrong ND, Srinivasasainagendra V, Patki A, et al. Genetic Contributors of Incident Stroke in 10,700 African Americans With Hypertension: A Meta-Analysis From the Genetics of Hypertension Associated Treatments and Reasons for Geographic and Racial Differences in Stroke Studies. *Front Genet*. 2021;12:781451. doi:10.3389/fgene.2021.781451

2. Parcha V, Malla G, Irvin MR, et al. Association of Transthyretin Val122Ile Variant With Incident Heart Failure Among Black Individuals. *JAMA*. Apr 12 2022;327(14):1368-1378. doi:10.1001/jama.2022.2896

3. Das S, Forer L, Schonherr S, et al. Next-generation genotype imputation service and methods. *Nat Genet*. Oct 2016;48(10):1284-1287. doi:10.1038/ng.3656

4. Price AL, Patterson NJ, Plenge RM, Weinblatt ME, Shadick NA, Reich D. Principal components analysis corrects for stratification in genome-wide association studies. *Nat Genet*. Aug 2006;38(8):904-9. doi:10.1038/ng1847

5. Chang CC, Chow CC, Tellier LC, Vattikuti S, Purcell SM, Lee JJ. Second-generation PLINK: rising to the challenge of larger and richer datasets. *Gigascience*. 2015;4:7. doi:10.1186/s13742-015-0047-8

© 2023 Yu H et al. *JAMA Network Open.*

eFigure 1. Study Participant Selection

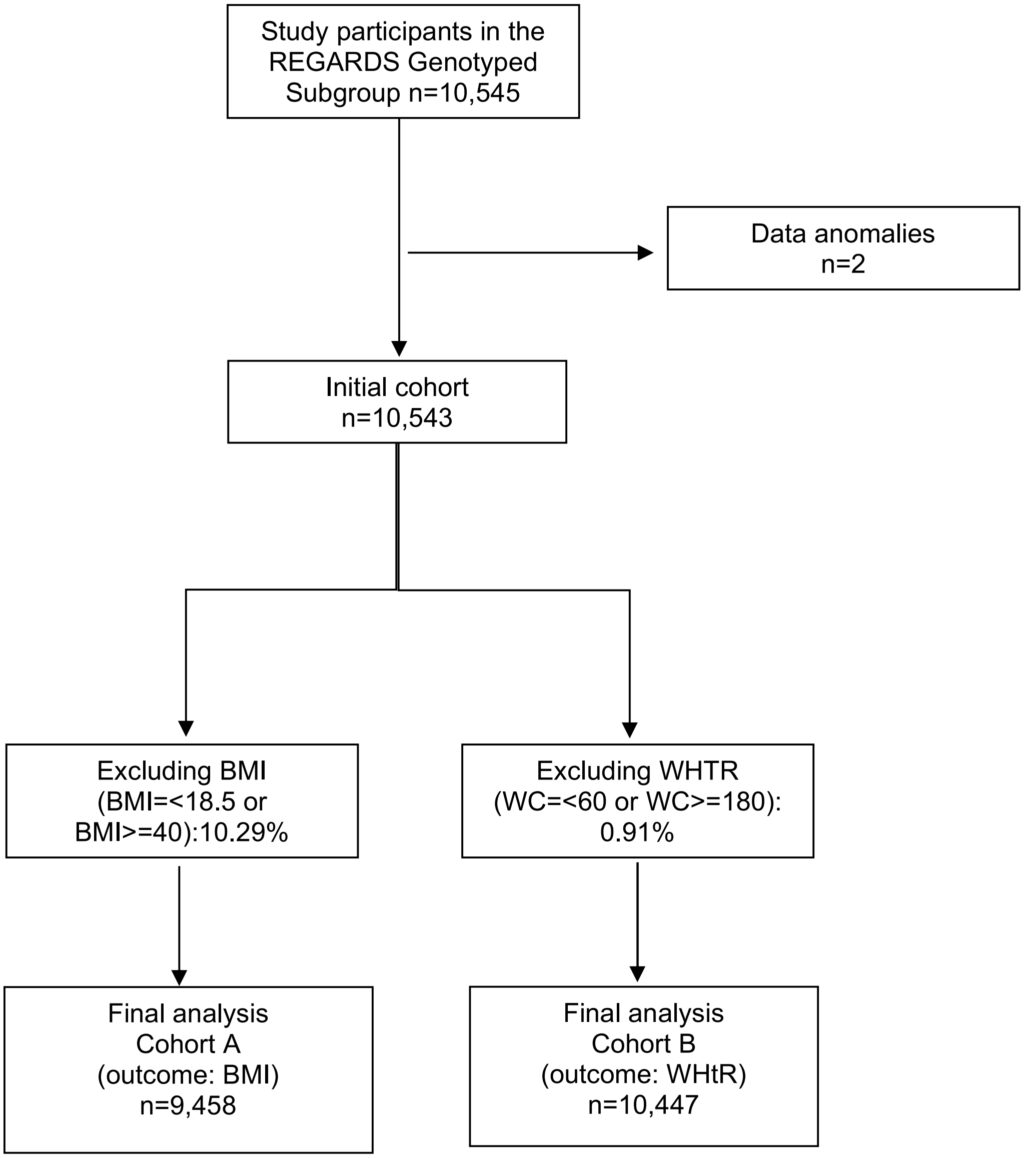

© 2023 Yu H et al. *JAMA Network Open.*

eTable 2. Comparisons of Effect Allele Frequencies of *FTO* SNVs Between Self-Identified Race Groups
(Cohort A: BMI, N=9,458; Cohort B: Waist to Height Ratio (WHtR), N=10,447).

**Cohort A:BMI analysis**			
***FTO* SNVs**	**Effect Allele**	Black N=7,832	White N=1,626
		Percent	Percent
rs1558902	A	11.10	40.43
rs1121980	A	47.26	42.68
rs17817449	G	39.38	40.01
rs8050136	A	43.62	40.01
rs9935401	A	43.95	40.03
rs3751812	T	10.80	39.51
rs9936385	C	47.73	40.04
rs9939609	A	47.72	40.01
rs9941349	T	18.72	41.36
rs9930506	G	21.18	43.30
rs9922708	T	21.11	43.39
**Cohort B:WHtR analysis**			
***FTO* SNVs**		Black N=8,743	White N=1,704
		Percent	Percent
rs1558902	A	11.17	40.29
rs1121980	A	47.25	42.55
rs17817449	G	39.40	39.88
rs8050136	A	43.6	39.91
rs9935401	A	40.00	39.94
rs3751812	T	10.85	39.38
rs9936385	C	47.75	39.94
rs9939609	A	47.74	39.91
rs9941349	T	18.84	41.20
rs9930506	G	21.18	43.30
rs9922708	T	21.26	43.08

© 2023 Yu H et al. *JAMA Network Open.*

eTable 3. Comparisons of Baseline Age, Income, Education Levels, Depression Score, and Stress Score Between Sex and Race Groups for BMI Analysis
(Cohort A, N=9,458, excluding cases analysis by analysis for Body Mass Index).

	Race				ANOVA		Sex				ANOVA	
**Age (years)**	Black<br>n=7,832		White<br>n=1,626				Female<br>n=5,276		Male<br>n=4,182			
	M	SD	M	SD	F	*p* †	M	SD	M	SD	F	*p*
Range	63.91<br>45-94	9.23	68.6<br>45-98	10.39	336.289	<0.001	64.4<br>45-94	9.71	65.2<br>45-98	9.47	8.250,	<0.001
**Depression score**	Black<br>n=7,621		White<br>n=1,588				Female<br>n=5,133		Male<br>n=4,076			
	M	SD	M	SD	F	*p* †	M	SD	M	SD	F	*p* †
Range	1.32<br>0-12	2.18	1.06<br>0-12	1.96	23.656	<0.001	1.51<br>0-12	2.33	.98<br>0-12	1.85	138.31,	<0.001
**Perceived Stress score**	Black<br>n=7,665		White<br>n=1,598				Female<br>n=5,164		Male<br>n=4,099			
	M	SD	M	SD	F	*p* †	M	SD	M	SD	F	*p* †
Range	5.82<br>0-16	2.65	5.47<br>0-16	2.31	20.530	<0.001	6.06<br>0-16	2.63	5.39<br>0-16	2.50	153.50,	<0.001
	**Race**				**Chi-Square**		**Sex**				**Chi-Square**	
**Education**	Black<br>n=7,824		White<br>n=1,624				Female<br>n=5,269		Male<br>n=4,179			
	Count	Percent	Count	Percent	*p*		Count	Percent	Count	Percent	*p*	
Less than high school	1389	17.8	139	8.6	<0.001		864	16.4	664	15.9	0.043	
High school	2143	27.4	386	23.8			1446	27.4	1083	25.9		
Some college	2116	27.0	457	28.1			1449	27.5	1124	26.9		
College graduate & above	2176	27.8	642	39.5			1510	28.7	1308	31.3		
**Income**	Black<br>n=6,905		White<br>n=1,421				Female<br>n=4,544		Male<br>n=3,782			
	Count	Percent	Count	Percent	*p*		Count	Percent	Count	Percent	*p*	
Below $20K	1926	27.9	222	15.6	<0.001		1442	31.7	706	18.7	<0.001	
20K-34K	2081	30.1	383	27.0			1393	30.7	1071	28.3		
35K-74K	2134	30.9	540	38.0			1316	29.0	1358	35.9		
75K & above	764	11.1	276	19.4			393	8.6	647	17.1		

† Welch tests performed for groups with unequal variances based on results from Levene's tests.

© 2023 Yu H et al. *JAMA Network Open.*

eTable 4. Comparisons of Baseline Age, Income, Education Levels, Depression Score, and Stress Score Between Sex and Self-Identified Race Groups for Waist-to-Height Ratio Analysis
(Cohort B, N=10,447, excluding cases analysis by analysis for Waist to Height Ratio).

	**Race**				**ANOVA**		**Sex**				**ANOVA**	
	Black n=8,743		White n=1,704				Female n=5,276		Male n=4,182			
	M	SD	M	SD	F	*p* †	M	SD	M	SD	F	*p*
**Age (years)**	63.6	9.19	68.4	10.35	377.337	<0.001	63.9	9.59	65.0	9.48	33.241	<0.001
Range	45-96		45-98				45-96		45-98			
	Black n=8,492		White n=1,664				Female n=5,869		Male n=4,287			
	M	SD	M	SD	F	*p* †	M	SD	M	SD	F	*p* †
**Depression score**	1.4	2.21	1.1	1.98	22.604	<0.001	1.5	2.36	1.0	1.87	155.090	<0.001
Range	0-12		0-12				0-12		0-12			
	Black n=8,549		White n=1,675				Female n=5,908		Male n=4,316			
	M	SD	M	SD	F	*p* †	M	SD	M	SD	F	*p* †
**Perceived Stress score**	5.8	2.65	5.5	2.31	23.492	<0.001	6.07	2.63	1.54	2.36	165.669	<0.001
Range	0-16		0-16				0-16		0-16			
	**Race**				**Chi-Square**		**Sex**				**Chi-Square**	
	Black n=7,824		White n=1,624				Female n=5,269		Male n=4,179			
	Count	Percent	Count	Percent	*p*		Count	Percent	Count	Percent	*p*	
**Education**					<0.001						0.043	
Less than high school	1570	18.0	153	9.0			1017	16.8	706	16.0		
High school	2410	27.6	407	23.9			1667	27.6	1150	26.1		
Some college	2385	27.3	479	28.1			1680	27.8	1184	41.3		
College graduate & above	2370	27.1	663	39.0			1672	27.7	1361	30.9		
	Black n=6,905		White n=1,421				Female n=4,544		Male n=3,782			
	Count	Percent	Count	Percent	*p*		Count	Percent	Count	Percent	*p*	
**Income**					<0.001						<0.001	
Below $20K	2216	24.1	241	16.2			1697	32.6	760	19.1		
20K-34K	2319	30.1	403	27.1			1599	30.8	1123	28.2		
35K-74K	2342	30.4	562	37.8			1475	28.4	1429	35.9		
75K & above	820	10.7	282	19.0			428	8.2	674	16.9		

† Welch tests performed for groups with unequal variances based on results from Levene's tests.

© 2023 Yu H et al. *JAMA Network Open.*

eTable 5. Results of Linkage Disequilibrium Among the 11 *FTO* SNVs in Self-Identified White Participants

**FTO SNVs**	rs1558902	rs1121980	rs17817449	rs8050136	rs9935401	rs3751812	rs9936385	rs9939609	rs9941349	rs9930506	rs9922708
**rs1558902**	1	0.9067	0.9421	0.9370	0.9382	0.9590	0.9357	0.9344	0.8836	0.8169	0.8136
**rs1121980**	0.9067	1	0.8938	0.8913	0.8900	0.8717	0.8875	0.8888	0.9425	0.8724	0.8688
**rs17817449**	0.9421	0.8938	1	0.994	0.9935	0.9729	0.9909	0.9922	0.9078	0.8387	0.8354
**rs8050136**	0.9370	0.8913	0.9948	1	0.9987	0.9729	0.9961	0.9974	0.9078	0.8411	0.8378
**rs9935401**	0.9383	0.8900	0.9935	0.9987	1	0.9742	0.9974	0.9961	0.9065	0.8398	0.8365
**rs3751812**	0.9590	0.8717	0.9729	0.9729	0.9742	1	0.9768	0.9755	0.9226	0.8541	0.8508
**rs9936385**	0.9357	0.8875	0.9909	0.9961	0.9974	0.9768	1	0.9987	0.9090	0.8423	0.8389
**rs9939609**	0.9344	0.8888	0.9922	0.9974	0.9961	0.9755	0.9987	1	0.9103	0.8436	0.8403
**rs9941349**	0.8836	0.9425	0.9078	0.9078	0.9065	0.9226	0.9090	0.9103	1	0.9260	0.9224
**rs9930506**	0.8169	0.8724	0.8387	0.8411	0.8398	0.8541	0.8423	0.8436	0.9260	1	0.9961
**rs9922708**	0.8136	0.8688	0.8354	0.8378	0.8365	0.8508	0.8389	0.8403	0.9224	0.9961	1

© 2023 Yu H et al. *JAMA Network Open.*

eTable 6. Results of Linkage Disequilibrium Among the 11 *FTO* SNVs in Self-Identified Black Participants

**FTO SNVs**	rs1558902	rs1121980	rs17817449	rs8050136	rs9935401	rs3751812	rs9936385	rs9939609	rs9941349	rs9930506	rs9922708
**rs1558902**	1	0.1328	0.1739	0.1441	0.1429	0.9638	0.1214	0.1211	0.4955	0.4170	0.4190
**rs1121980**	0.1328	1	0.7206	0.7307	0.7206	0.1326	0.5951	0.5949	0.1476	0.1800	0.1792
**rs17817449**	0.1739	0.7206	1	0.8374	0.8254	0.1836	0.7073	0.7071	0.0411	0.0210	0.0216
**rs8050136**	0.1441	0.7307	0.8374	1	0.9852	0.1537	0.8441	0.8445	0.0623	0.0938	0.0935
**rs9935401**	0.1429	0.7206	0.8254	0.9852	1	0.1521	0.8539	0.8534	0.0608	0.0925	0.0915
**rs3751812**	0.9638	0.1326	0.1836	0.1537	0.1521	1	0.1305	0.1301	0.5192	0.4385	0.4405
**rs9936385**	0.1214	0.5950	0.7073	0.8441	0.8539	0.1305	1	0.9995	0.0435	0.0691	0.0683
**rs9939609**	0.1211	0.5949	0.7071	0.8445	0.8534	0.1301	0.9995	1	0.0436	0.0692	0.0684
**rs9941349**	0.4955	0.1476	0.0411	0.0623	0.0608	0.5192	0.0435	0.0436	1	0.7839	0.7867
**rs9930506**	0.4170	0.1800	0.0210	0.0938	0.0925	0.4385	0.0691	0.0692	0.7839	1	0.9958
**rs9922708**	0.4190	0.1792	0.0216	0.0935	0.0915	0.4405	0.068	0.0684	0.7867	0.9958	1
**LD Block – first SNP from each LD block listed (r^2>0.8) used as proxy denoted with ***											
**LD Block 1**	rs1558902*	rs3751812									
**LD Block 2**	rs1121980*										
**LD Block 3**	rs17817449*										
**LD Block 4**	rs8050136*	rs9935401	rs9936385	rs9939609							
**LD Block 5**	rs9941349*										
**LD Block 6**	rs9930506*	rs9922708									

© 2023 Yu H et al. *JAMA Network Open.*

eTable 7. Associations Between BMI and WHtR by FTO Proxy SNVs, Controlling for Sex (Females as the Reference Group) in Self-Identified Black Participants or White Participants
For Black participants, Bonferroni corrected significance level at 0.00833+; for White participants, Bonferroni corrected significance level at 0.05*

Black participants	BMI as a dependent variable †		WHtR as a dependent variable†	
	FTO	Sex	FTO	Sex
	P	P	P	P
**rs1558902**	0.004+	<.001	0.015	<.001
**rs1121980**	0.104	<.001	0.666	<.001
**rs17817449**	0.307	<.001	0.814	<.001
**rs8050136**	0.340	<.001	0.449	<.001
**rs9941349***	0.006+	<.001	0.078	<.001
**rs9930506***	0.022	<.001	0.041	<.001
**White participants**	**BMI as a dependent variable†**		**WHtR as a dependent variable†**	
	FTO	Sex	FTO	Sex
	P	P	P	P
**rs1558902**	0.0020*	0.1057	0.0186*	0.1106

† General linear model: Obesity indicator = intercept + FTO + sex + PC 1-10 + error

© 2023 Yu H et al. *JAMA Network Open.*

eTable 8. Interaction Terms Between FTO SNVs, Sex, and Age in Black Participants, Controlling for Psychosocial Status (Lower Education Levels and Lower Income Levels as the Reference Group) (outcome: BMI/WHtR, Bonferroni corrected significance level at 0.00833).

**BMI†**	**FTO* Sex**		**FTO* Age**	
	Beta	P	Beta	P
rs1558902	0.151	0.561	-0.022	0.134
rs1121980	-0.299	0.077	-0.012	0.188
rs17817449	-0.280	0.104	-0.017	0.077
rs8050136	-0.252	0.138	-0.019	0.042
rs9941349	0.151	0.277	-0.011	0.299
rs9930506	0.169	0.405	-0.011	0.337
**WHtR†**	**FTO* Sex**		**FTO*Age**	
	Beta	P	Beta	P
rs1558902	0.006	0.173	0.000	0.899
rs1121980	-0.002	0.410	0.000	0.766
rs17817449	0.001	0.642	0.000	0.310
rs8050136	-0.002	0.550	0.000	0.336
rs9941349	0.004	0.270	0.000	0.290
rs9930506	0.003	0.160	0.000	0.747

† General linear model: Obesity indicator (BMI) = intercept +FTO SNP LD Block) + sex + FTO SNP* Sex (or FTO* Age) + age + income + education + stress + depression + PC 1-10 + error

† General linear model: Obesity indicator (WHtR) = intercept + FTO SNP LD Block + sex + FTO SNP* Sex (or FTO* Age) + age + income + education + stress + depression + PC 1-10 + error

© 2023 Yu H et al. *JAMA Network Open.*

eTable 9. Significant Interaction Terms Between *FTO* SNV rs1558902:A, Sex, and Age in White Participants, Controlling for Psychosocial Status (Lower Education Levels and Lower Income Levels as the Reference Group) (outcome: BMI/WHtR, Bonferroni corrected significance level at 0.05).

**BMI†**	**FTO* Sex**		**FTO* Age**	
	Beta	P	Beta	P
**LD Block 1 (rs1558902:A)**	0.781	<0.001	-0.057	<0.001
**WHtR†**	**FTO* Sex**		**FTO* Age**	
	Beta	P	Beta	P
**LD Block 1 (rs1558902:A)**	0.018	<0.001	-0.001	0.017

† General linear model: Obesity indicator (BMI) = intercept +FTO SNP + sex + FTO SNP* Sex (or FTO* Age) + age + income + education + stress + depression + PC 1-10 + error

† General linear model: Obesity indicator (WHtR) = intercept + FTO SNP + sex + FTO SNP* Sex (or FTO* Age) + age + income + education + stress + depression + PC 1-10 + error

© 2023 Yu H et al. *JAMA Network Open.*

eFigure 2. Mediation Model of Obesity Indicators (BMI) in Relation With 6 *FTO* LD Block Proxy SNVs and Heart Disease in Black Participants
(Bonferroni corrected significance level = 0.00833).

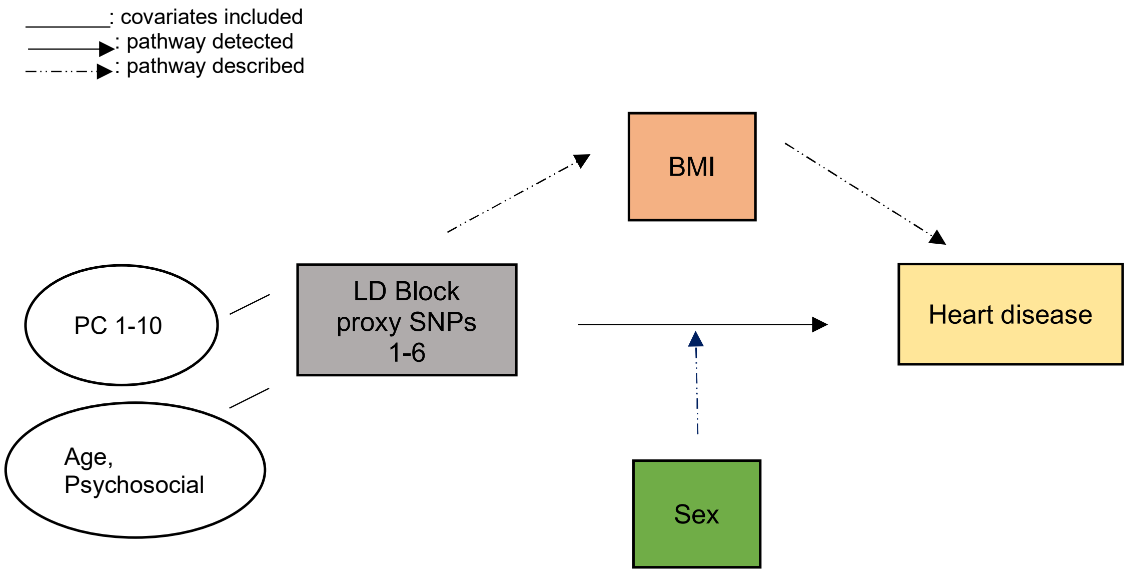

© 2023 Yu H et al. *JAMA Network Open.*

eTable 10. Interaction Terms Between Genetic Susceptibility by *FTO* SNV rs1121980: A and Sex in Black Participants
(outcome: heart disease, no heart disease as the reference group, Bonferroni corrected significance level at 0.00833). Others tested were not significant (data not shown).

**Heart disease †**	FTO* Sex	
	Coeff.	p
**LD Block 2**<br>**rs1121980: A**	0.030	0.760

† Binary logistic model: heart disease= intercept + FTO SNPs + sex + FTO SNPs* Sex + age + income + education + stress + depression + PC 1-10 + error

© 2023 Yu H et al. *JAMA Network Open.*

eFigure 3. Mediation Model of Obesity Indicators (BMI and WHtR) in Relation With FTO SNVs, and Hypertension/Diabetes in White Participants (significance level = 0.05).

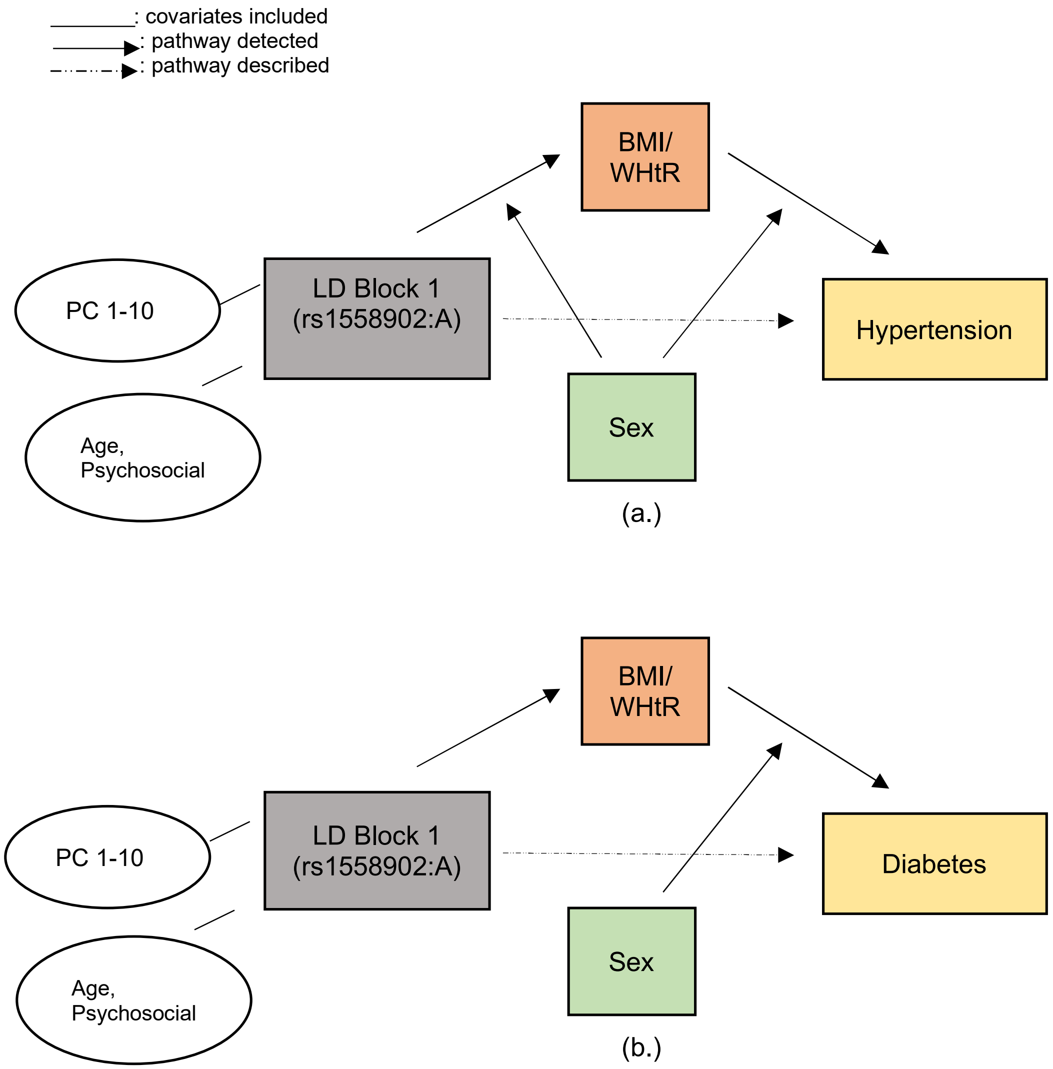

© 2023 Yu H et al. *JAMA Network Open.*

eTable 11. Results of Mediation and Moderation analyses of BMI/WHtR, LD Block 1 (rs1558902:A), and Diabetes (No Diabetes as the Reference Group) in White Participants

**Model 3**								
		**Consequent**						
		**M (BMI)**			**Y (Diabetes)**			
**Antecedent**		Coeff.	SE	p		Coeff.	SE	p
X LD Block 1 (rs1558902:A)	a	0.4762	0.1768	0.0072	C’	0.0753	0.1093	0.491
M (BMI)		-	-	-	b	0.1382	0.0171	<0.001
Constant	i_m	33.4239	1.5708	<0.001	i_y	-5.2693	1.1713	<0.001
		R^2= 0.0615, F=4.9830, p<0.001				R^2= 0.1220, p<0.001		
**Moderation**		Coeff.	SE	p		Coeff.	SE	95% CI
W (sex)		-	-	-	W*M female	0.0689	0.0288	0.0193 - 0.1309
					male	0.0633	0.0264	0.0187 - 0.1208
						Index = - 0.0056		
**Model 4**								
		**Consequent**						
		**M (WHtR)**			**Y (Diabetes)**			
**Antecedent**		Coeff.	SE	p		Coeff.	SE	p
X LD Block 1 (rs1558902:A)	a	0.0062	0.0028	0.0289	C’	0.0706	0.1112	0.4940
M (BMI)		-	-	-	b	14.6063	3.7939	<0.001
Constant	i_m	0.5696	0.0253	<0.001	i_y	-9.0536	2.5021	<0.001
		R^2=0.0519, F=4.1580, p<0.001				R^2=0.1593, p<0.001		
**Moderation**		Coeff.	SE	p		Coeff.	SE	95% CI
W (sex)		-	-	-	W*M female	0.0751	0.0389	0.0053 - 0.1594
					male	0.0595	0.0313	0.0041 - 0.1289
						Index = -0.0156		

† Indirect effect= a*b; Direct effect= C’; Total effect= a*b + C’

© 2023 Yu H et al. *JAMA Network Open.*
