# Supplementary material for: Sex and Race Differences in Obesity-Related Genetic Susceptibility and Risk of Cardiometabolic Disease in Older US Adults
Source: JAMA Netw Open. 2023 Dec 8;6(12):e2347171. doi: 10.1001/jamanetworkopen.2023.47171 (PMC10709778; doi:10.1001/jamanetworkopen.2023.47171)
Supplement: Supplement 2. — Data Sharing Statement [file jamanetwopen-e2347171-s002.pdf]

Data Sharing Statement

Yu. Sex and Race Differences in Obesity-Related Genetic Susceptibility and Risk of Cardiometabolic Disease in Older US Adults. *JAMA Netw Open*. Published December 11, 2023. doi:10.1001/jamanetworkopen.2023.47171

Data
Data available: Yes
Data types: Deidentified participant data, Data (not involving human participants), Data dictionary
How to access data: nmda@uab.edu or kakaiser@uab.edu
When available: With publication

Supporting Documents
Document types: Informed consent form
How to access documents: http://www.regardsstudy.org.
When available: With publication

Additional Information
Who can access the data: The data will be made available to researchers whose proposed use of the data has been approved.
Types of analyses: The data will be made available for a specified purpose.
Mechanisms of data availability: The data will be made available after approval of a proposal.
